# Gene-Transcript Expression in Urine Supernatant and Urine Cell-Sediment Are Different but Equally Useful for Detecting Prostate Cancer

**DOI:** 10.3390/cancers15030789

**Published:** 2023-01-27

**Authors:** Marcelino Yazbek Hanna, Mark Winterbone, Shea P. O’Connell, Mireia Olivan, Rachel Hurst, Rob Mills, Colin S. Cooper, Daniel S. Brewer, Jeremy Clark

**Affiliations:** 1 Urology Department Castle Hill, Hull University Teaching Hospital, Castle Rd, Cottingham HU16 5JQ, UK; 2 Norwich Medical School, University of East Anglia, Norwich NR4 7TJ, UK; 3 Translational Oncology Laboratory, Department of Pathology and Experimental Therapy, School of Medicine, Universitat de Barcelona, 08907 L’Hospitalet de Llobregat, Spain; 4 Program of Molecular Mechanisms and Experimental Therapy in Oncology (ONCOBELL), Bellvitge Biomedical Research Institute (IDIBELL), 08908 L’Hospitalet de Llobregat, Spain; 5 Norfolk and Norwich University Hospitals NHS Foundation Trust, Norwich NR4 7UY, UK; 6 Earlham Institute, Norwich NR4 7UZ, UK

**Keywords:** urine, prostate, cancer, biomarker, extracellular vesicles, cell-sediment

## Abstract

**Simple Summary:**

Cancer cells and vesicles are transported in prostatic secretions to the urethra and are flushed out on urination. These cells and vesicles contain prostate-specific gene transcripts, but their relative usefulness in prostate cancer detection has not been fully determined. We have examined the expression of 167 gene-probes in vesicle and cell fractions from 76 urine samples provided by men with and without prostate cancer. Measured gene expression profiles varied between the fractions. Many genes were useful as biomarkers for PCa in one fraction only, supporting the analysis of fractionated urine over the analysis of whole urine. Signatures constructed from cell or vesicle data were equally good at distinguishing prostate cancer from no-cancer controls. A combined-fraction signature did not show significant improvement. We present data on the relative expression of six housekeeping genes and the potential tissue origin of cells and vesicles in urine.

**Abstract:**

There is considerable interest in urine as a non-invasive liquid biopsy to detect prostate cancer (PCa). PCa-specific transcripts such as the *TMPRSS2:ERG* fusion gene can be found in both urine extracellular vesicles (EVs) and urine cell-sediment (Cell) but the relative usefulness of these and other genes in each fraction in PCa detection has not been fully elucidated. Urine samples from 76 men (PCa *n* = 40, non-cancer *n* = 36) were analysed by NanoString for 154 PCa-associated genes-probes, 11 tissue-specific, and six housekeeping. Comparison to qRT-PCR data for four genes (*PCA3*, *OR51E2*, *FOLH1*, and *RPLP2*) was strong (*r* = 0.51–0.95, Spearman *p* < 0.00001). Comparing EV to Cells, differential gene expression analysis found 57 gene-probes significantly more highly expressed in 100 ng of amplified cDNA products from the EV fraction, and 26 in Cells (*p* < 0.05; edgeR). Expression levels of prostate-specific genes (*KLK2*, *KLK3*) measured were ~20× higher in EVs, while PTPRC (white-blood Cells) was ~1000× higher in Cells. Boruta analysis identified 11 gene-probes as useful in detecting PCa: two were useful in both fractions (*PCA3*, *HOXC6*), five in EVs alone (*GJB1*, *RPS10*, *TMPRSS2:ERG*, *ERG*_Exons_4-5, *HPN*) and four from Cell (*ERG*_Exons_6-7, *OR51E2*, *SPINK1*, *IMPDH2*), suggesting that it is beneficial to fractionate whole urine prior to analysis. The five housekeeping genes were not significantly differentially expressed between PCa and non-cancer samples. Expression signatures from Cell, EV and combined data did not show evidence for one fraction providing superior information over the other.

## 1. Introduction

Prostate cancer (PCa) is the second most commonly diagnosed cancer in men in the world [1]. Suspicion of PCa is based on serum PSA testing, abnormal digital rectal examination (DRE) and more recently MRI [2]. Confirmation of PCa is by needle biopsy, an invasive procedure that can have significant morbidity. Improving the pre-screening methods used to decide who to biopsy would reduce costs and patient stress. The use of ‘liquid biopsy’ tests using samples of blood, saliva and urine have been investigated. Blood is used to examine PSA levels and also to detect circulating tumor cells [3] and cell-free nucleic acids, though dilution of markers in the large volume of circulating blood has made sensitivity an issue [4]. Saliva has been utilised to examine germline changes such as faulty DNA-repair genes that could result in a predisposition for cancer development. Urine in comparison is used to examine the presence or absence of prostate cancer within a prostate, and the connection of the prostate to the urinary system presents several advantages in PCa detection. The prostate is a secretory organ that drains into the urethra. Prostate cancers shed cells and extracellular vesicles (EVs) which are carried with these secretions and are flushed out of the body on urination in the first 15 mL of urine [5,6]. Urine, therefore, represents an attractive non-invasive liquid-biopsy source of PCa-biomarkers. 

Urine studies have largely focussed on urine cell-sediment in which mRNA transcripts such as *PCA3* [7] and *TMPRSS2:ERG* [8] have shown diagnostic utility. The use of cell-free RNA harvested from EVs in urine supernatant is a promising alternative. EVs contain PCa-specific transcripts and EV membranes have been shown to protect from nucleases and other potentially harmful chemicals present in urine [9]. We have recently used EV expression data for 38 gene-probes to construct Prostate Urine Risk (PUR) signatures, which have shown the potential to predict disease progression over a five-year follow-up period (HR = 8.2) [10]. Only a few studies to date have attempted to compare cell-sediment and EV urine fractions, each only examining a handful of genes, with no consensus on each fraction’s potential to differentiate between PCa and non-PCa. Dijkstra et al. [11] compared levels of *PCA3* and *TMPRSS2-ERG* in the cell-sediments and EVs from 30 men scheduled for a biopsy. 10% of cell-sediments were unusable due to the formation of crystals following centrifugation whilst none were lost in the EV fraction. Dijkstra recorded higher yields of RNA from cell-sediments than EVs and when using *PCA3* mRNA levels better diagnostic utility in the cell-sediments fraction was observed. In contrast, Pellegrini et al. [12] found that the EV fraction had higher total RNA yields (*n* = 105), better RNA quality as assessed by RIN-score and higher levels of *PCA3* and *ERG* RNAs (*n* = 52). Hendriks and colleagues [13] compared the expression of mRNA transcripts in whole urine, cell-sediment and EVs (*n* = 29). They observed that expression of *KLK3*, *PCA3* and *ERG* were highest in whole urine, followed by EV, and lowest in cell-sediments. They reported that *PCA3* transcripts were expressed significantly more highly in PCa patients compared to non-PCa in both the whole-urine and cell fractions but not in the EV fractions, while *ERG* was only significantly differentially expressed in the cell-sediment fraction. These studies suggest that, although urine EVs may provide a more robust source of biomarkers, the cell-sediment fraction appears to have greater diagnostic utility, albeit in only four gene transcripts examined. 

Could combining the examination of transcripts in both Cell-sediment (Cell) and EVs improve the utility of urine biomarkers to detect prostate cancer? To examine this question, NanoString data from 167 gene-probes were interrogated (including the 38 PUR signature gene-probes) in Cell and EV fractions from 76 samples and correlated with PCa disease status on biopsy. 

## 2. Materials and Methods

### 2.1. Clinical Cohort

Post-DRE urine samples were collected from 90 men attending the Norfolk and Norwich University Hospitals. Ethical approval for the study was gained from the East of England Research Ethics Committee, UK (ref 12/EE/0058). Men were divided by PCa status: Men with PCa on 10-core trans-rectal ultrasound-guided (TRUS) biopsy, and ‘Non-Cancer’ (NC), which consisted of 7 unbiopsied men with a normal PSA for age [14] and 29 men with a raised serum PSA (≥4 ng/mL) that were found to be negative for cancer on TRUS biopsy (see Table 1 for cohort clinical characteristics). 

### 2.2. Cell and EV Fractions

Urine samples were centrifuged to pellet the cells, EVs were harvested from the supernatant by filtration using a 100 kDa spin-filter unit (Millipore, Burlington, MA, USA), and RNA was extracted from each fraction using RNeasy Micro columns (Qiagen #74004, Hilden, Germany) as in Connell et al. 2019 [10].

### 2.3. NanoString Data: Feature Selection and Analysis

Cell and EV RNA samples (5–20 ng) were amplified with the Ovation PicoSL WTA System V2 kit (Nugen #3312-48, Leeds, UK) and NanoString analysis for 167 gene-probes was performed on 100 ng amplified cDNA products at the Human Dendritic Cell Laboratory, Newcastle University, UK as described by Connell et al., [10] (see Table S1 for the 167 NanoString gene-probes used). Where multiple probes per transcript were used, the exact exons targeted are stated, e.g., *ERG_Exons_4-5* and *ERG_Exons_6-7.* NanoString data were subject to quality control prior to normalisation as per NanoString’s guidelines. Fourteen samples were removed due to NanoString normalisation factors being outside the manufacturer’s acceptable range (less than 0.1 or greater than 10.0, https://nanostring.com/support-documents/gene-expression-data-analysis-guidelines/ (accessed on 1 January 2022)). The measured expression levels are the counts for that probe detected in 100 ng of amplified cDNA products and should be considered as a proportion rather than the absolute total amount of RNA for that gene in a particular urine fraction.

All analyses were performed in R version 3.4.1. The *edgeR* package was used to pre-process and examine differential gene expression within and between the Cell and EV urine fractions (data in Table S2). *EdgeR* pre-processing implements the filtering strategy described by Chen et al. (2016) [15] and which retained genes that had a minimum of 10 counts in 5 samples, leaving 105/167 gene-probes for subsequent analysis. Biological variation across gene-probes was estimated based on the use of negative binomial distribution and generalised linear models [16,17].

Gene-probes useful in identifying PCa were selected by comparing Non-Cancer samples to PCa-samples. A robust feature selection workflow was implemented that used the Boruta algorithm [16] and bootstrap resampling as described in Connell et al. (2020) [17]. Boruta uses a random forest algorithm iteratively compared feature importance against randomly shuffled predictors named “shadow features”. Features that performed significantly worse compared to the best-performing shadow feature (Shadow Max) at each permutation were consecutively dropped until only stable features remained. Gene-probes were identified as ‘tentative’ or ‘confirmed’ by comparison to the performance of the shadow features. ‘Confirmed’ indicates that a gene probe performed statistically better than the maximum performance of the shadow feature (ShadowMax)—this is a high threshold as the ShadowMax could easily be quite high by chance. ‘Tentative’ indicates that a gene probe performed significantly better than the mean performance of the Shadow gene-probe (ShadowMean) but was not statistically better than the ShadowMax. 

The Boruta-selected gene-probes were combined in a random forest model to produce three risk scores for prostate cancer using data from (i) the Cell-sediment fraction, (ii) the EV fraction, and (iii) a combined risk score using data from both fractions.

### 2.4. RT-PCR Analysis

Quantitative RT-PCR (qRT-PCR) was performed for *PCA3*, *OR51E2*, *FOLH1*, *KLK3*, and *RPLP2* following the protocol of Sequeiros et al. [18] (see Table S3 for PCR primers). qRT-PCR analysis of the samples was performed on a 384-well plate. Duplicate qRT-PCRs were run on a separate 384-well plate on the same day. The presence of *TMPRSS2:ERG* transcripts was detected using *TMPRSS2* exon1 and *ERG* exon 6 primers as in Clark et al. [19]. For comparison of qRT-PCR data with NanoString data for the same genes, we used Cohen’s (1988) conventions to interpret effect size; small/weak: *r* ≥ 0.1, moderate: *r* ≥ 0.3, large/strong: *r* ≥ 0.5 [20]. 

## 3. Results

### 3.1. Gene-Transcript Expression in Urine EV and Urine Cell-Sediment Are Different

#### 3.1.1. No Differences Were Observed in the Expression of Housekeeper Genes between Cancer and Non-Cancer in Both Urine Fractions

**Figure 1:**
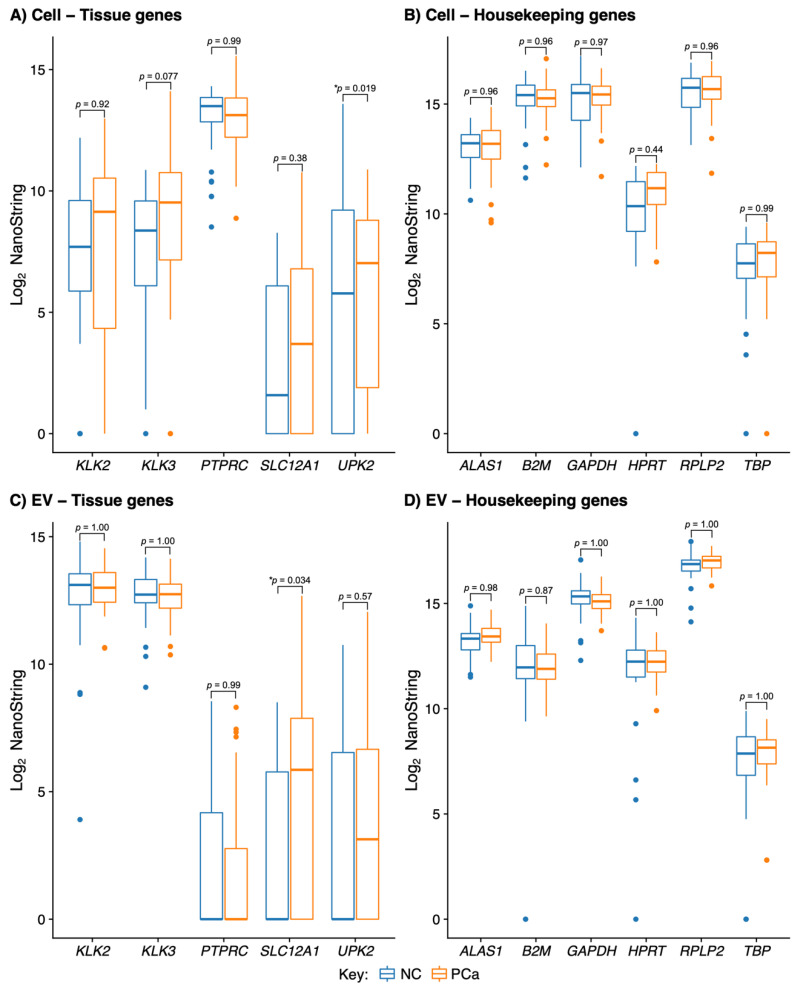
NanoString signals for 5 tissue-specific gene-probes (**A**,**C**) and 6 housekeeping gene-probes (**B**,**D**) in urine cell-sediment (Cell, upper panel) and urine extracellular vesicles (EV, lower panel). Blue indicates non-cancer samples (NC), and red indicates prostate cancer samples (PCa). *p*-values are for statistical difference between cancer and non-cancer by edgeR; * indicates a significant difference (False Discovery Rate *p* < 0.05).

We analysed Cell and EV fraction samples from urine using a 167-probe custom-built NanoString assay which contained six housekeeping gene-probes, five tissue-specific genes and 154 PCa-associated gene-probes (Table S1). No significant differences in housekeeper expression (*ALAS1*, *B2M*, *GAPDH*, *HPRT*, *RPLP2*, *TBP*) were found between non-cancer (NC) and prostate cancer (PCa) samples in either fraction (False Discovery Rate (FDR) *p* < 0.05; edgeR; Figure 1B,D; Table S2).

#### 3.1.2. EV and Cell Fractions Have a Different Profile of Tissue of Origin

To investigate the origin of the Cells and EVs found in urine, we analysed five tissue-specific gene-probes corresponding to the following tissue/cell types: normal prostate (*KLK2*, *KLK3*), bladder (*UPK2)*, kidney (*SLC12A1)* and blood leukocytes (*PTPRC*) (Figure 1A,C). Median *KLK2* and *KLK3* expression levels measured in 100 ng of amplified cDNA products were ~20-fold higher in EV compared to the Cell fraction. *PTPRC*, a gene expressed in all nucleated cells of hematopoietic origin, was detected at a high value in the Cell fraction with only minimal levels of expression in the EVs (median > 1000-fold lower). Measured levels of bladder-specific *UPK2* and kidney-specific *SLC12A1* were low in both fractions. Differences between Non-Cancer (NC, *n* = 36) and PCa (*n* = 40) were only significant for *SLC12A1* in EVs (median levels ~50-fold higher in the PCa EV samples, FDR *p* = 0.034; edgeR; Table S2).

#### 3.1.3. Most Gene-Probes Are Differentially Expressed between EV and Cell Fractions

After pre-filtering for present probes (*n* = 105; Table S1), 83% of gene-probes were significantly differentially expressed in 100 ng of amplified cDNA products between the EV and Cell fractions (FDR *p* < 0.05; edgeR; Table 2 and Table S2). Of these, 57 were found to be significantly more highly expressed in the EV fraction and 26 were more highly expressed in the Cell fraction. 

#### 3.1.4. Expression Changes between Non-Cancer and Cancer Are Different in the EV and Cell Fractions

**Figure 2:**
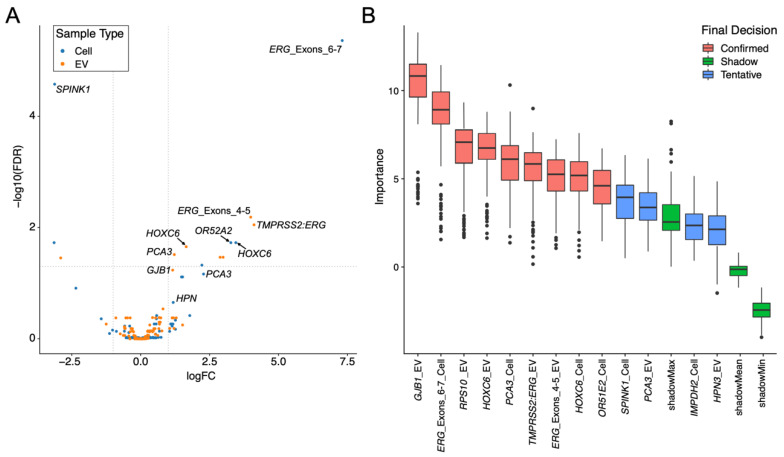
NanoString Gene-probe expression for cancer and non-cancer samples in urine cell-sediment (Cell) and extracellular vesicle (EV) samples. (**A**) Volcano plot, dashed lines are thresholds for significance (horizontal) and fold-changes (vertical). Genes selected by Boruta analysis are indicated. (**B**) Boruta selection of potentially useful gene-probes in prostate cancer detection. The fraction that the gene-probe was found to be useful in is indicated. Red indicates a gene-probe in the indicated urine fraction was significantly better than the ShadowMax feature (‘confirmed’); blue indicates the gene data was significantly better than the ShadowMean (‘tentative’ see main text).

Thirteen probes/fraction combinations were significantly differentially expressed between NC and PCa in the Cell and EV expression data (FDR *p* < 0.05; edgeR; Table 3; data presented as a Volcano plot in Figure 2A)—7 in EVs and 6 in Cell; all were overexpressed in cancer apart from *CDKN3*, *SPINK1* and *UPK2*. Three commonly used urine biomarker genes were in the top 10 differentially expressed gene/fraction combinations: *ERG*, *HOXC6*, and *PCA3*. *ERG* and *HOXC6* were significantly more highly expressed in PCa in both Cell and EVs fractions, while *PCA3* was only significantly higher in PCa in EVs. Median expression levels of these three genes were higher in EV than Cell fractions (EV vs. Cell: *ERG* 46 vs. 0.5; *HOXC6* 1432 vs. 7.5; *PCA3* 2750 vs. 163).

### 3.2. Expression Levels from RT-PCR and NanoString Are Strongly Correlated for Both EV and Cell Urine Fractions

Quantitative RT-PCR (qRT-PCR) was used to verify the expression of five NanoString gene-probes: (i) *KLK3* a prostate-specific gene used for normalisation in the Progensa *PCA3* test [38]; (ii) *RPLP2*, a gene used for data normalisation in construction of the PUR signatures [10]; three commonly used PCa-related genes: (iii) *PCA3* (selected in Boruta analysis multivariate analysis for association with PCa—see below), (iv) *OR51E2* (aka *PSGR* Prostate-Specific G-Protein Coupled Receptor) (Boruta selected) and (v) *FOLH1* (aka PSMA, prostate-specific membrane antigen).

qRT-PCR Ct values for *RPLP2*, *FOLH1*, *OR51E2*, *PCA3* and *KLK3* were compared to the NanoString expression signals for these genes in 71 EV samples and 66 Cell samples (Figure S2). A strong correlation for *RPLP2*, *FOLH1*, *OR51E2* and *PCA3* was observed for both EV (Spearman correlation coefficient *r* > 0.6, *p* < 0.00001; Table 4) and Cell (*r* > 0.6, *p* < 0.00001; Table 4). 

The *KLK3* data was more complex and a group of 13 samples (7xNC, 6xPCa) had low *KLK3* qRT-PCR/High Ct values in both EV and Cell fractions. When all the data were included, there was a strong correlation in Cell (*r* = 0.70, *p* < 0.00001) but in EV samples the correlation was weaker (*r* = 0.51, *p* = 0.0017) (See Discussion). Correlation of *KLK3* NanoString and RT-PCR data without these 13 samples provided *r* values of >0.85 for both fractions.

Non-quantitative RT-PCR analysis was performed for the presence/absence of *TMPRSS2:ERG* fusion gene transcripts using *TMPRSS2* exon 1 forward and *ERG* exon 6 reverse primers. 14/21 PCa samples were positive for *TMPRSS2:ERG* by PCR in the EV fraction and 10/21 in the Cell fraction. The RT-PCR *TMPRSS2:ERG* status was significantly associated with the NanoString *TMPRSS2:ERG* values (*p* = 4.36 × 10^−5^ (EV); *p* = 1.25 × 10^−4^ (Cell); Mann-Whitney U test). In NC samples, RT-PCR also detected a *TMPRSS2:ERG* in 9/29 EV samples and 6/23 Cell samples. The data for NanoString probes *ERG*_Exon_6-7 and *ERG*_Exon_4-5 showed similar associations to RT-PCR positivity in both EV and Cell fractions (*ERG*_Exon_6-7: *p* = 1.14 × 10^−5^ (EV); *p* = 1.51 × 10^−7^ (Cell); *ERG*_Exon_4-5: *p* = 3.40 × 10^−6^ (EV); *p* = 0.0113 (Cell); Mann-Whitney U test). Nearly half (48%) of PCa EV samples were triple-positive for all three NanoString probes, while only 22% of Cell samples were triple-positive. Six of the 36 non-cancer samples (17%) were triple positive by EV but none were triple positive in Cell samples. All the triple-positive NC EV samples were from patients with a raised PSA and a PCa-negative-TRUS biopsy.

### 3.3. Each Urine Fraction Has Different Genes That Are Important in Predicting the Presence of Prostate Cancer

**Figure 3:**
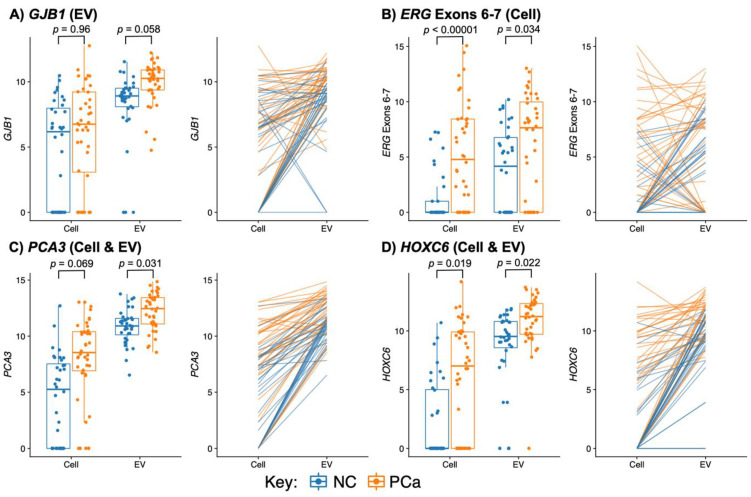
Box plots of Boruta-selected NanoString gene-probe data from urine cell sediment (Cell) and urine extracellular vesicle (EV) RNA in men with prostate cancer (PCa, orange) and controls with no evidence of cancer (NC, blue, see Methods). Orange (PCa) and blue (NC) lines link NanoString expression data for paired Cell and EV samples from individual urine samples. *p*-values (False Discovery Rate) are for the statistical difference between cancer and non-cancer by edgeR. Gene-probes were: (**A**) *GJB1*, (**B**) *ERG*_Exons_6-7, (**C**) *PCA3*, (**D**) *HOXC6*.

The Boruta algorithm [16] was used to identify the importance of gene-probes in predicting the presence of PCa on biopsy. Thirteen gene-probe/urine fraction combinations were identified as performing significantly better than the mean performance of the Shadow gene-probe (ShadowMean) (see methods, Figure 2B). Nine of these gene-probe/urine fraction combinations were statistically better than the maximum performance of the Shadow feature (ShadowMax) and as such were deemed ‘confirmed’. These nine gene-probe/urine fraction combinations corresponded to eight gene-probes providing readout from six genes (*GJB1*, *PCA3*, *HOXC6*, *OR51E2*, *RPS10*, *TMPRSS2:ERG)*. Expression data for four examples are presented in Figure 3 (see Figure S1 for all Boruta-selected gene-probes).

#### 3.3.1. PCA3 and HOXC6 Were Useful in Both EV and Cell Sediment Fractions

Two gene-probes were identified as being useful in both Cell and EV fractions: *HOXC6* and *PCA3*.

*PCA3* (Prostate Cancer Associated 3, Figure 3C) was a potentially useful feature in both fractions, albeit tentatively in the EV fraction. In the Cells, 36% of the NC samples had no expression of *PCA3* compared to 8% of the PCa samples. The median expression of *PCA3* in PCa v NC samples was 8.6-fold higher in the Cell fraction and 2.6-fold higher in the EV. *PCA3* is a prostate-tissue-specific, noncoding messenger RNA [39] overexpressed in urine cell sediment in 95% of men with PCa [7]. A *PCA3* assay has been developed using *PCA3* expression in urine cell-sediment (Progensa^®^; Gen-Probe, San Diego, CA, USA). The *PCA3* test has been approved by the FDA as a diagnostic test only in the setting of an initial negative prostate biopsy to predict the presence of PCa on a second biopsy [7]. The PCA3 test has not been approved for use in the National Health Service in the UK [2] and the European Association of Urology has stated that its impact at a single-patient level remains highly questionable [40]. In tissue, *PCA3* has a bimodal distribution in both biopsy and radical prostatectomy (RP) samples, where low *PCA3* expression was significantly associated with high grade disease (*p* < 0.001). *PCA3* had a poor performance in predicting high grade disease in initial biopsy tissue (GS ≥ 8) with 55% sensitivity and high false negative rates. In excised prostates, low *PCA3* is also associated with adverse pathological features, clinical recurrence outcome and a greater probability of metastatic progression (*p* < 0.001) [41]. In meta-analyses of PCA3-test studies of patients with previous negative biopsies, an AUC of 0.739 (with a *PCA3* score cut-off of >35) and an AUC of 0.63 were found for PCa on a second biopsy [42]. Meta-analyses of the urine test by Luo et al. (2014) concluded that results were heterogeneous (sensitivity: 0.75–0.93; specificity 0.44–0.78) [43]. 

*HOXC6* (Homeobox C6, Figure 3D) had much higher expression levels in EV than Cell (median 1432 vs. median 8). In EV, *HOXC6* was expressed by >95% of the samples with median expression 3⋅2-fold higher in the PCa samples than in NC. In the Cell fraction, *HOXC6* was detected in 68% of the PCa samples compared to 36% of the NC samples. *HOXC6* is overexpressed in primary, metastasized and castration-resistant PCa, and expression was not influenced by androgens or treatments targeting the AR signaling pathway [33]. Silencing of *HOXC6* expression using small-interfering RNA (siRNA) resulted in decreased proliferation rates for both androgen-dependent LnCaP cells and the LnCaP- derived androgen-independent C4-2 cell line, and induced apoptosis [44]. *HOXC6* mRNA levels are higher in the urine cell-sediment of PCa patients [33], and patients with high *HOXC6* expression had shorter overall survival than those with low *HOXC6* expression [45]. *HOXC6* is used in the SelectMDx prostate cancer urine test alongside *DLX1*. SelectMDx has been found to underperform when compared to template biopsy [46] and mpMRI [47] in the detection of clinically significant PCa.

#### 3.3.2. EV Fraction Genes Useful for Prostate Cancer Detection

Five gene-probes were useful in the EV fraction only: *GJB1*, *RPS10*, *TMPRSS2:ERG*, *ERG*_Exons_4-5, and *HPN*. Data for *TMPRSS2:ERG* and the two *ERG* probes are presented in Section 3.3.4.

*GJB1* (Gap Junction Protein Beta 1) expression in EVs (Figure 3A) was identified with the highest importance in discerning PCa from NC. *GJB1* expression in EVs was a median of 2.5-fold lower in the NC samples compared to the PCa samples and 70% (25/36) of the NC samples had expression below the lowest quartile of the PCa samples. In contrast, expression of *GJB1* in the Cell was not significantly different in cancer and non-cancer samples. *GJB1* has been associated with PCa [48] and has been identified as a prognostic marker for renal cancer [49]. *GJB1* is a member of the gap junction connexin family of proteins that regulates and controls the transfer of communication signals across cell membranes, primarily in the liver and peripheral nervous system. Expression levels of GJB1 protein (aka Connexin 32, CX32) were found to be the same in PCa and benign prostatic hyperplasia samples [50]. No publications for the use of *GJB1* in urine tests were found apart from one publication by our group (Connell et al., 2020 [17]).

*RPS10* (Ribosomal Protein S10) was highly expressed in both fractions and no samples were negative. It was identified as being useful in detecting PCa within the EV fraction with *RPS10* expression levels decreased in cancer (Figure S1). *RPS10* has been found to be overexpressed at the protein level in PCa [51]. We have not found any reports suggesting the use of *RPS10* as a urinary biomarker.

*HPN* (Hepsin) was tentatively identified as being potentially useful for PCa-detection and therefore would require further testing in a larger cohort. It encodes a type II transmembrane serine protease involved in diverse cellular functions, including the maintenance of cell morphology. HPN is upregulated in PCa and correlates with disease progression [52].

#### 3.3.3. Cellular Genes Useful for PCa Detection

Four gene-probes were useful in the Cell fraction only: *ERG*_Exons_6-7, *OR51E2*, *SPINK1* and *IMPDH2*.

*OR51E2* (Olfactory Receptor Family 51 Subfamily E Member 2, Figure S1) was 30-fold more highly expressed in the EV fraction compared to the Cell fraction (median 2006 vs. 63). However, *OR51E2* was only useful for detecting PCa in the Cell fraction, in which NanoString signal was above the threshold in only 33% of NC compared to 73% of PCa, with a median differential expression of 127-fold. *OR51E2* has been found to be overexpressed in PCa tissue [53] and in the urine cell-sediment of men with PCa [34].

*IMPDH2* followed a similar pattern of expression to *OR51E2*, being higher in EVs but more informative in the Cell fraction for PCa detection. *IMPDH2* (Inosine Monophosphate Dehydrogenase 2) encodes the rate-limiting enzyme in the de novo guanine nucleotide biosynthesis required for DNA and RNA synthesis. Increased serum levels of *IMPDH2* were significantly associated with Gleason ≥8 PCa, suggesting its potential as a serological tumor marker [54]. *IMPDH2*, in our study, was identified as being potentially useful but only tentatively and would require further testing in a larger cohort. 

*SPINK1* (Serine Peptidase Inhibitor Kazal Type 1) was the only Boruta-selected probe that had a higher median expression in Cell than EVs (~2-fold). *SPINK1* has been reported to be overexpressed in a group of *ETS*-fusion negative PCa and *SPINK1*-positive PCa was reported to be an aggressive PCa subtype [36]. Laxman et al. [55] demonstrated an increase in *SPINK1* in PCa and suggested its use in a multiplex assay using urinary sediments.

#### 3.3.4. TMPRSS2:ERG and ERG Probes Are Useful Biomarkers in Urine

A *TMPRSS2:ERG* translocation is detectable in ~50% of all prostate cancer foci [33,56], and results in overexpression of *ERG* (ETS Transcription Factor ERG) from exon 4 to 3′-end in >95% of TMPRSS2:ERG cases [57]. An increased copy number of *TMPRSS2:ERG* has been associated with a worse prognosis [56]. Three NanoString probes were designed to detect transcripts from a *TMPRSS2:ERG* fusion gene: a *TMPRSS2:ERG* gene probe spanning the most commonly found *TMPRSS2*_exon1/*ERG*_exon4 fusion transcripts [57] and two probes to *ERG* sequences that lay 3’ to the usual gene translocation point, one spanning exons 4 to 5 (*ERG*_Exon4-5), and the other spanning exons 6 to 7 (*ERG*_Exon_6-7). All three *ERG* probes were found to be useful for PCa detection: *ERG*_Exons_6-7 levels in Cell (Figure 3) and EV levels of *ERG*_Exons_4-5 and *TMPRSS2:ERG* fusion (Figure S1). EV expression levels for the *TMPRSS2:ERG* and *ERG_*Exons_4-5 probes in PCa samples were similar to each other and were ~4.5 times higher than probe signals in the Cells. Median signals for the *ERG*_Exon_6-7 probe were much higher in both fractions than those obtained from the *TMPRSS2:ERG* and *ERG*_Exon_4-5 probes: ~2.5-fold higher in the EV, and 6.5-fold higher in the Cell fractions (see Figure S2 and Discussion). In the PCa EV samples, the three probes—‘*TMPRSS2:ERG*’, ‘*ERG*_Exon_4-5’ and ‘*ERG*_Exon_6-7’ probe—were above the threshold in 22/40, 23/40 and 26/40 PCa samples, respectively and 95% of the *TMPRSS2:ERG*-positive samples were positive for *ERG*_Exon_4-5 and 90% for *ERG*_Exon_6-7, with 19/40 PCa samples triple-positive for all three probes in the EV fraction. For the Cell PCa samples, the three probes had lower rates of detection (11/40, 10/40, 23/40) with 81% *ERG*_Exon_4-5 and 100% *ERG*_Exon_6-7 in concurrence with the *TMPRSS2:ERG* probe positive samples. In addition, 9/40 PCa samples were triple-positive in the Cell fraction. For the NC samples, 6/36 were triple positive in the EV fraction, but none were triple positive in the Cell fraction. All the triple-positive NC samples had a raised PSA (>4 ng/mL) but were negative for PCa on biopsy. 

Due to the multifocal nature of PCa, tumor foci can be found with and without a *TMPRSS2:ERG* in individual prostates [58] such that they are present in ~70% of cancerous prostates [59], making them a more useful biomarker than was originally apparent. Young et al. determined that *TMPRSS2:ERG* urine transcript levels aided *PCA3* in predicting the presence of PCa and correlated with *ERG* expression in PCa tissue [60]. Tomlins et al. combined the detection of *TMPRSS2:ERG* fusion transcripts and *PCA3* with serum PSA levels and the result from the multivariate Prostate Cancer Prevention Trial risk calculator version 1.0 (PCPT-RC) in a combined predictor, which they called Mi-Prostate score (MiPS) [61]. MiPS had a significantly improved AUC for the detection of PCa and Gs ≥ 7 on biopsy when compared to PSA or PCPT-RC alone.

### 3.4. Gene-Transcript Expression in Urine EV and Urine Cell-Sediment Are Equally Useful for Detecting Prostate Cancer

**Figure 4:**
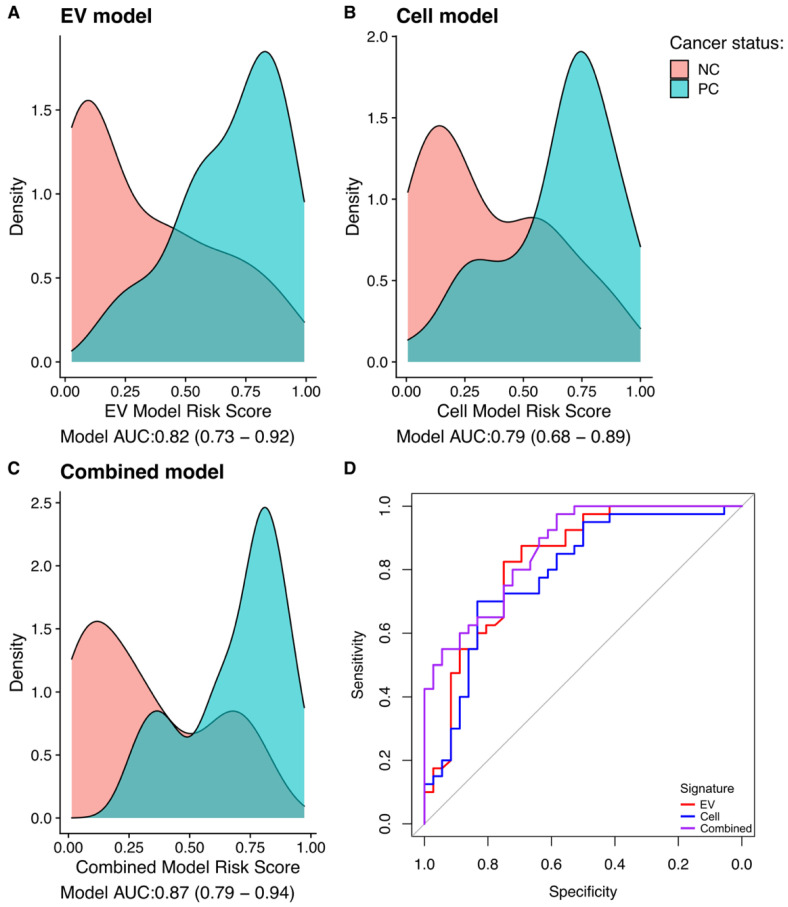
Model Metrics. (**A**–**C**): Density plots for the EC, Cell, and combined signatures (models). Red is non-cancer and blue is prostate cancer. (**D**): AUC analysis for the three signatures: red = EV, blue = Cell, purple = combined signature.

A random forests model to predict cancer status was built incorporating the gene-probes identified by Boruta analysis for the samples in each fraction (Section 3.2); in addition, an optimal predictor was produced for the EV and Cell fractions combined (Table S4). The output for each model was a diagnostic risk score. Each of the three signatures were able to predict the presence of cancer, with the area under the curve values (AUCs) being significantly better than a random predictor. AUCs for the three models were: EV signature AUC 0.82 (bootstrap Confidence Interval 0.729–0.921), Cell signature 0.79 (0.684–0.894), Combined signature (0.87 (0.788–0.944), Figure 4D). The combined model had the highest AUC, which was within the range of the confidence intervals for the other signatures and so there was no evidence for a significant improvement. Density plots for the three models were constructed (Figure 4A–C), each signature showing distinct peaks for NC and PCa. 

## 4. Discussion

We have examined the transcriptomes of urine EVs and cell-sediment from 76 men with a large number of gene-probes (*n* = 167). We have compared the relative expression of these gene-probes in 100 ng of amplified cDNA products from each fraction and investigated their usefulness in Pca detection. Thirteen gene-probe/urine fraction combinations were identified as being potentially useful in the identification of prostate cancer. *GJB1* expression in EVs was found to be the strongest candidate. Five gene-probes were solely useful in the EV fraction and four gene-probes were solely useful in the cell-sediment, suggesting that fractionation prior to analysis can provide more potential biomarkers. Only *PCA3* and *HOXC6* were useful in both fractions. Three models were constructed from the EV, Cell and combined EV & Cell data which showed a strong separation of PCa and non-cancer samples. 

The vast majority of the NanoString probes used here were designed for gene transcripts reported to be differentially expressed in PCa tissue [10]. It was therefore surprising that the bulk of the 154 PCa-linked gene probes did not show any useful association with cancer in urine cell-sediment or EV RNA. The potential reasons for this are different for each fraction. In cell sediment, PCa cells are a tiny minority [62], data which is supported by the very strong expression of the nucleated-blood-cell gene PTPRC in urine cells by Quek [63] and by our data here in both PCa and non-cancer urine samples. We and Pellegini et al. [12] observed a much higher level of expression of prostate-specific genes (*KLK2* and *KLK3*) in the EV fraction than the Cell fraction (*KLK2* 22-fold higher, *KLK3* 50-fold higher) indicating an enrichment for prostate-specific transcripts within the EV fraction compared to the cell sediments (Figure 1). 

Boruta analysis of the two urinary fractions identified 13 gene-probe/urine fraction combinations as being potentially useful in identifying prostate cancer. These selected genes included *PCA3*, *HOXC6* and *TMPRSS2:ERG*, that have previously been identified as having utility as urinary biomarkers for PCa [61,64,65,66]. Two of these genes (*PCA3* and *HOXC6*) appear to be useful in both the Cell and EV fractions, though the Boruta importance score was higher for the EV fraction. Expression was observed in almost all EV samples (Figure 3) in comparison to for example *HOXC6* which had limited expression in the NC samples being detected in only 14 of 35 samples (39%) compared to 28 of 40 (70%) in the PCa groups. The gene identified as having the most predictive utility was *GJB1* in the EV fraction (Figure 2). *GJB1* was highly expressed by almost all the samples in the EV fraction with a higher median gene expression in the PCa-associated groups. Three NanoString probes were designed to detect *TMPRSS2:ERG* fusion transcripts or overexpression of *ERG* as a result of the translocation [19,31]. The robustness of detecting *TMPRSS2:ERG* in samples was very different between the EV and Cell fractions and there was a lack of sensitivity for the detection of *TMPRSS2:ERG* in cell-sediment, which may reflect the low levels of PCa cells in this fraction. Patients with a PCa-negative 10-core TRUS biopsy have been reported to harbour undiscovered PCa in around 20% of cases [67]. The *ERG*_Exons_6-7 probe appeared to have a much higher sensitivity of detection than the other two probes, with *ERG*_Exons_6-7 detecting raised expression of *ERG* in 23 Cell samples compared to *ERG*_Exons_4-5 (*n* = 10) or *TMPRSS2:ERG* (*n* = 11). The reason for this may relate to the precise fusion transcripts generated or the extremely GC-rich nature of *TMPRSS2* exon1 sequences (79% GC). GC-rich regions have a much poorer efficiency of reverse transcription [68] due to RNA secondary structure which could result in a lower detection rate for the *TMPRSS2* sequences and the immediately adjacent *ERG*_Exons_4-5 sequence relative to the more distal *ERG* ex 6-7 sequences. The *ERG*_Exons_6-7 probe gave on average a 2.5-fold higher signal than the other two probes in EVs and a 6.5-fold higher signal in the Cell fractions of PCa samples. We hypothesise that it was this additional sensitivity of the *ERG*_Exons_6-7 probe that enabled it to have utility in the Cell fraction.

Comparison of qRT-PCR data with NanoString data for four genes displayed a strong correlation between the two methods indicating that NanoString is a useful method for multiplex gene expression analysis in agreement with previous studies [69]. In contrast, the correlation between expression values from NanoString and qRT-PCR for *KLK3* was poor, which was due to low expression values for *KLK3* by qRT-PCR in a subgroup of samples, a difference that was not detected by NanoString. Interestingly, the *KLK3* qRT-PCR values were low in both Cell and EV fractions of the same 13 urine samples. A possible explanation for this comes from David et al. 2002, who found that transcript splice variants of *KLK3* can include all or part of intron 1 [70]. Notably, the forward *KLK3* PCR primer used in our investigations spanned the exon1-2 boundary, with the two 3’ nucleotides being in exon 2. Thus, the presence of intron 1 sequences would make the transcripts un-PCRable with this primer set. The NanoString probes are much larger (2 × 50 nt) and reported *KLK3* levels in these samples were not discernible from the other samples. 

In our study presented here, three gene probes (*GJB1*, *RPS10* and *HOXC6*) provided comparable utility to *PCA3*. *GJB1* and *RPS10* have not to our knowledge been used in urine tests by other laboratories and could open up new avenues of research. Our data suggest that increasing the number of PCa-associated genes in a urine test should provide a more level playing field for the detection of cancers. Knowing which urine fraction to use for these multi-gene tests is critical. In addition, our results demonstrate that the exact probe sequences used to detect gene transcripts expressed by, for example, a *TMPRSS2:ERG* fusion gene is important. 

We identified different genes as useful biomarkers in different urinary fractions indicating the utility of using both fractions for biomarker development. Using whole urine is attractive due to the reduction in required preparation steps and Hendriks et al. [13] suggested that whole urine is a useful substrate, at least for *PCA3* and *ERG.* In our results, *PCA3* and *ERG* probes were highlighted as potential biomarkers in both the Cell and EV fractions. However, using whole urine for other novel markers may reduce the potential ability to detect PCa. For example, *OR51E2* is ~10-fold more highly expressed in EVs than the Cell fraction (Figure S1) but was only useful as a PCa-biomarker in the Cell fraction, therefore if the two fractions were combined it is likely that the high EV expression would obscure the difference in expression between the NC and PCa groups seen in the cell-sediments. Similarly, for *RPS10* the reduction in expression in the PCa groups of the EV fraction may be lost when combined with the expression from the Cell fraction. These data would suggest that screens for new PCa-biomarkers should be conducted on each individual fraction and that multiple probes for the detection of individual gene transcripts should be tested to optimise performance. An aspect not covered in this study is that of gene mutations, for example, mutations of mitochondrial genes associated with patient survival [71], and it may be fruitful to integrate targeted analysis of specific urine gene transcripts for mutations in future urine tests. We are currently in the validation phase of our urine research for which we have collected 2500 samples for analysis and are creating an accredited diagnostics laboratory that will enable us to provide urine results to patients.

## 5. Conclusions

We have interrogated urine Cell and EV RNA with 167 gene-probes and observed differential expression between fractions. We have identified 11 genes as useful in identifying PCa, which are distributed between the Cell and EV fractions, including the biomarker *GJB1*. These data indicate that a useful strategy for improving the identification of PCa through urinary biomarker analysis would involve the measurement of specific gene-targets from different urinary fractions.

## Figures and Tables

**Table 1:** Cohort characteristics. GG: Gleason Grade Group, No Bx: No Biopsy performed, Neg Bx: PCa-negative on biopsy, Age: median age in years (Y), IQR: interquartile range, N: Number of samples.

Characteristic	Non-Cancer	Prostate Cancer
Number of Samples	36	40
Age (IQR)	66 (12.3)	70.0 (9.5)
PSA (ng/mL) (IQR)	6.3 (4.0)	9.1 (5.4)
Biopsy results (N, %)	No Bx (7, 19%) Neg Bx (29, 79%)	GG1 (6, 26%) GG2 (17, 74%) GG3 (6, 35 %) GG ≥ 4 (11, 65%)

**Table 2:** The top 10 differentially expressed genes between EV and Cell fractions. Log FC: log2 fold change, *p* value: False discovery rate significance (edgeR). Summary information on gene function and published information linked to PCa is provided here, and more detailed information on each gene is provided in Table S1.

Gene Name	Log_2_ FC	*p*-Value	Expression	Gene Function/Link to PCa
*NEAT1*	−8.65	<0.00001	Lower in EVs	Bone metastasis [21]
*MIR4435.1HG lOC541471*	−4.43	<0.00001	Lower in EVs	No publications on PCa
*IFT57*	2.81	<0.00001	Higher in EVs	Pro-apoptotic [22]
*B2M*	−3.9	<0.00001	Lower in EVs	Housekeeper [23]
*BTG2*	−3.67	<0.00001	Lower in EVs	Tumor-suppressor [24]
*MCTP1*	−9.42	<0.00001	Lower in EVs	Calcium signaling [25]
*DPP4*	3.05	<0.00001	Higher in EVs	Overexpressed in PCa [26]
*APOC1*	−8.16	<0.00001	Lower in EVs	Overexpressed in PCa [27]
*H1.2*	1.68	<0.00001	Higher in EVs	Apoptotic response to DNA damage [28]
*ECI2*	2.09	<0.00001	Higher in EVs	Knock-out may have a therapeutic response in PCa [29]

**Table 3:** The significantly differentially expressed genes between non-Cancer and prostate cancer (PCa) in univariate analysis. Log FC: log^2^ fold change, *p* value: False discovery rate significance (edgeR). Summary information on gene function and published information linked to PCa is provided here; more detailed information on each gene is provided in Table S1.

Gene Name	Fraction	log FC	*p* Value	Expression	Gene Function/Link to Cancer
*CDKN3*	EVs	−2.9	0.0352	Lower in PCa	Overexpressed in PCa [30]
*ERG*_Exons*_*4-5	EVs	3.99	0.00650	Higher in PCa	Overexpression due to TMPRSS2:ERG translocation [31]
*ERG*_Exons*_*6-7	Cell	7.31	4.40 × 10^−6^	Higher in PCa	As above
*ERG*_Exons*_*6-7	EVs	2.88	0.0342	Higher in PCa	As above
*FOLH1*	Cell	2.22	0.0474	Higher in PCa	Overexpressed in PCa [32]
*HOXC6*	Cell	3.45	0.0187	Higher in PCa	Overexpressed in PCa urine sediment [33]
*HOXC6*	EVs	1.65	0.0221	Higher in PCa	Overexpressed in PCa urine sediment [33]
*OR51E2*	Cell	3.27	0.0187	Higher in PCa	Overexpressed in PCa urine sediment [34]
*PCA3*	EVs	1.22	0.0306	Higher in PCa	Overexpressed in PCa urine cell sediment [7]
*SLC12A1*	EVs	2.99	0.0342	Higher in PCa	Kidney-specific [35]
*SPINK1*	Cell	−3.12	0.0187	Lower in PCa	Overexpressed in TMPRSS2:ERG-negative PCa [36]
*TMPRSS2:ERG*	EVs	4.11	0.0893	Higher in PCa	Translocation in 50% of PCa [31]
*UPK2*	Cell	−3.14	0.0187	Lower in PCa	bladder-specific expression [37]

**Table 4:** Correlation results between qRT-PCR Ct values and NanoString expression signals.

Gene	Experiment	Spearman Correlation Coefficient	*p*-Value
*FOLH1*	Cells	0.71	<0.00001
*FOLH1*	EV	0.68	<0.00001
*KLK3*	Cells	0.7	<0.00001
*KLK3*	EV	0.51	<0.00001
*OR51E2*	Cells	0.77	<0.00001
*OR51E2*	EV	0.74	<0.00001
*PCA3*	Cells	0.88	<0.00001
*PCA3*	EV	0.95	<0.00001
*RPLP2*	Cells	0.86	<0.00001
*RPLP2*	EV	0.79	<0.00001

## Data Availability

Data is contained within the article or supplementary material.

## Supplementary Materials

The following supporting information can be downloaded at: https://www.mdpi.com/article/10.3390/cancers15030789/s1, Figure S1: NanoString expression data for Boruta selected genes; Figure S2: Comparison of NanoString data and qRT-PCR data for 5 gene-transcripts; Table S1: Nanostring Data; Table S2: Differential gene-expression analysis; Table S3: RT-PCR-Nanostring comparisons; Table S4: Boruta signatures.
