# Supplementary material for: Gene-Transcript Expression in Urine Supernatant and Urine Cell-Sediment Are Different but Equally Useful for Detecting Prostate Cancer
Source: Cancers (Basel). 2023 Jan 27;15(3):789. doi: 10.3390/cancers15030789 (PMC9913640; doi:10.3390/cancers15030789)
Supplement: Supplementary file 1 [file cancers-15-00789-s001.zip › Figures S1-S2.pdf]

Figure S1.
A) Box plots of Boruta-selected NanoString gene-probe data from urine cell sediment (Cell) and urine extracellular vesicle (EV) RNA in men with prostate cancer (PCa, orange) and controls with no evidence of cancer (NC, blue) (see Results and Methods).
B) Orange (PCa) and blue (NC) lines link NanoString expression data for paired Cell and EV samples from individual urine samples.

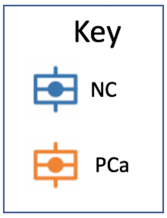

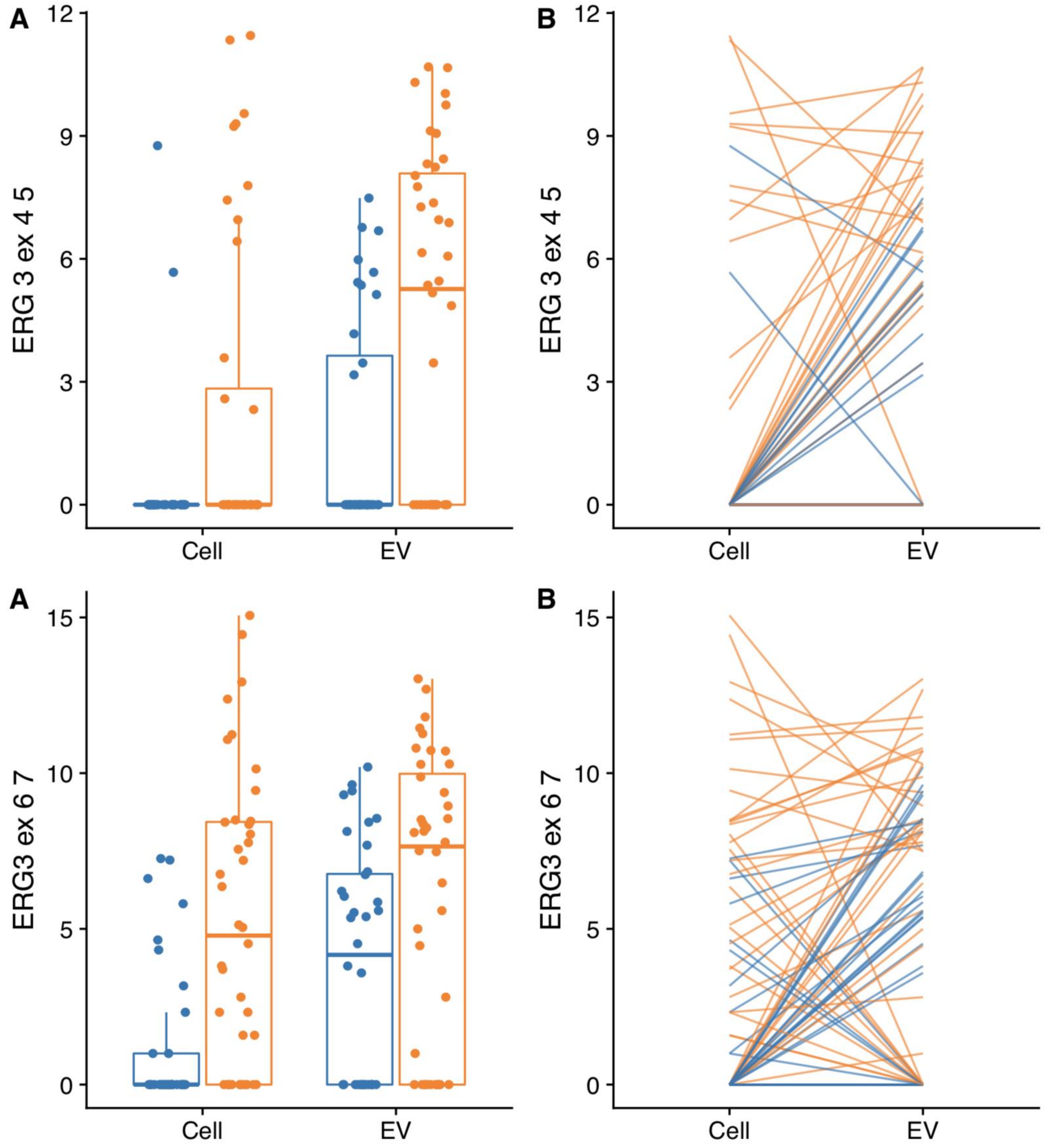

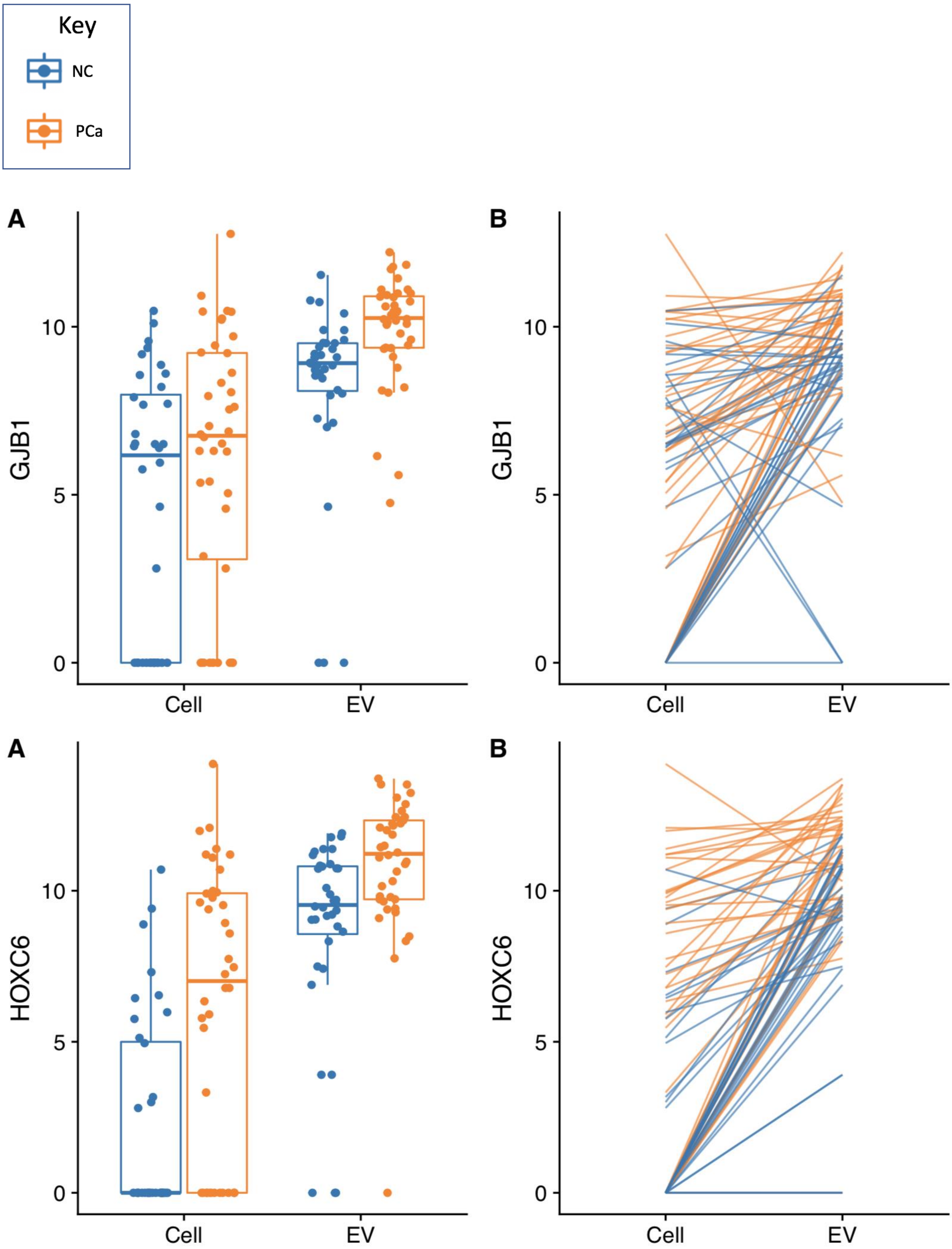
Key
NC
PCa
A
GJB1
10
5
0
Cell
EV
B
GJB1
10
5
0
Cell
EV
A
HOXC6
10
5
0
Cell
EV
B
HOXC6
10
5
0
Cell
EV

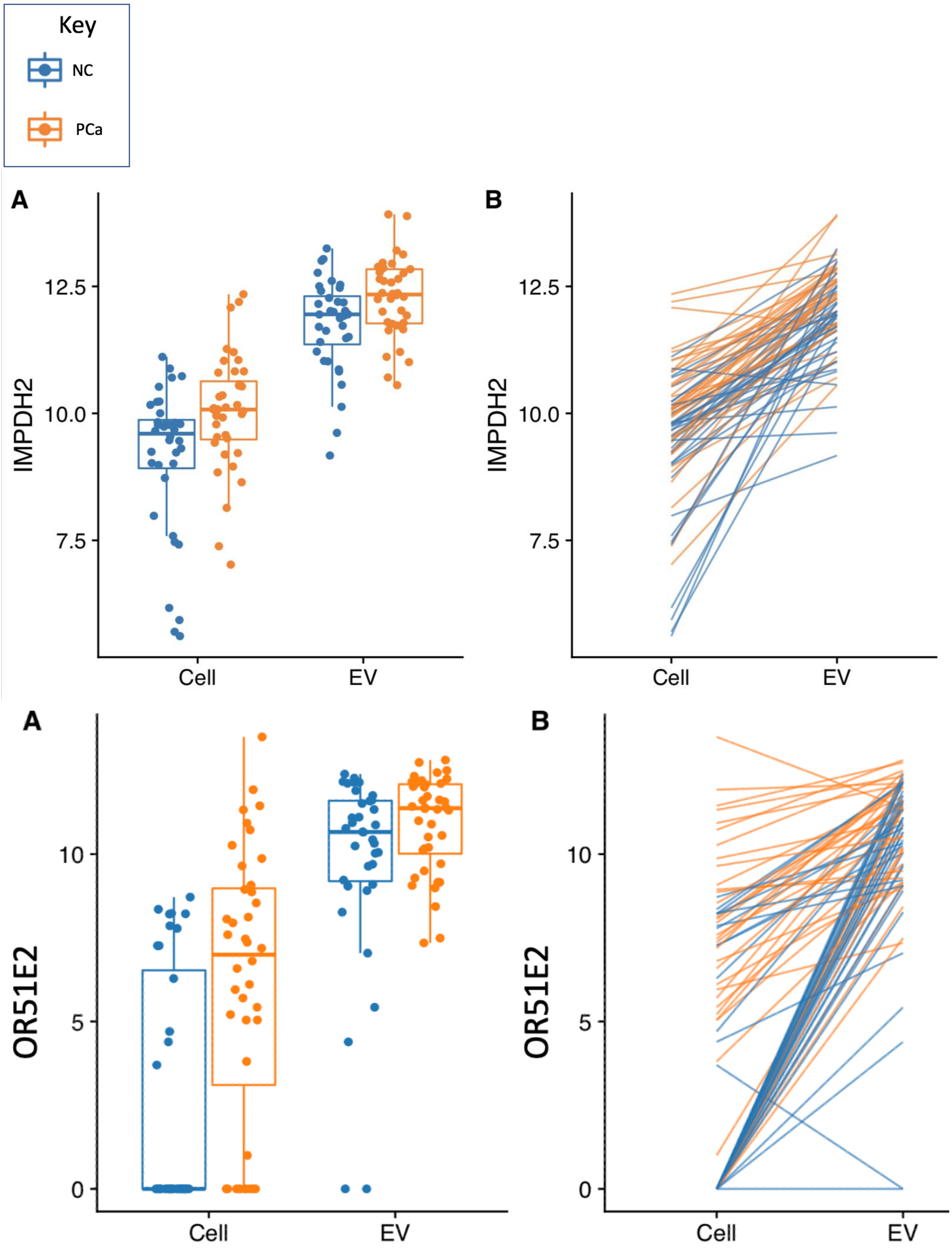

Key
NC
PCa
A
B
IMPDH2
12.5
10.0
7.5
Cell
EV
A
B
OR51E2
10
5
0
Cell
EV

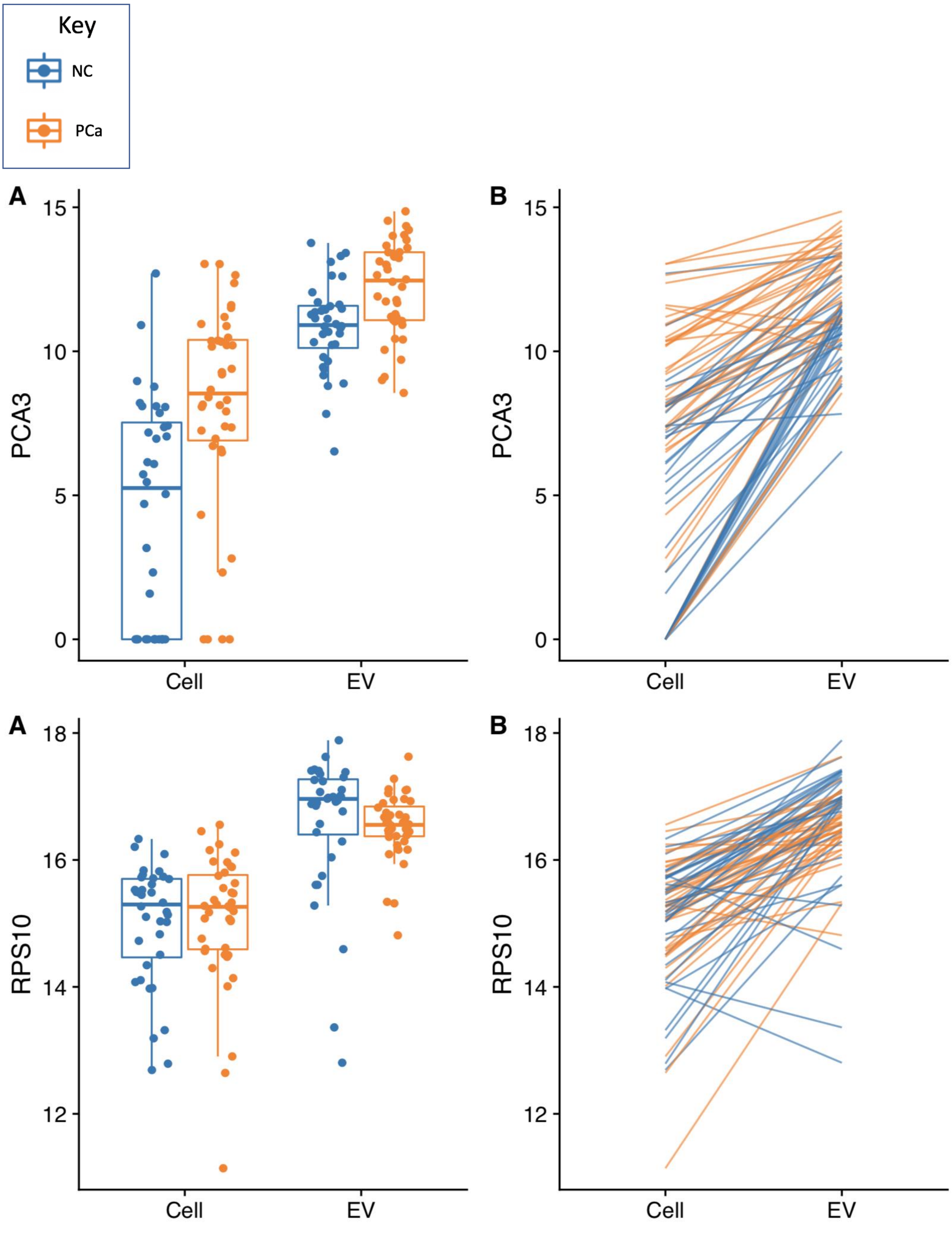
Key
NC
PCa
A
B
PCA3
15
10
5
0
Cell
EV
RPS10
18
16
14
12

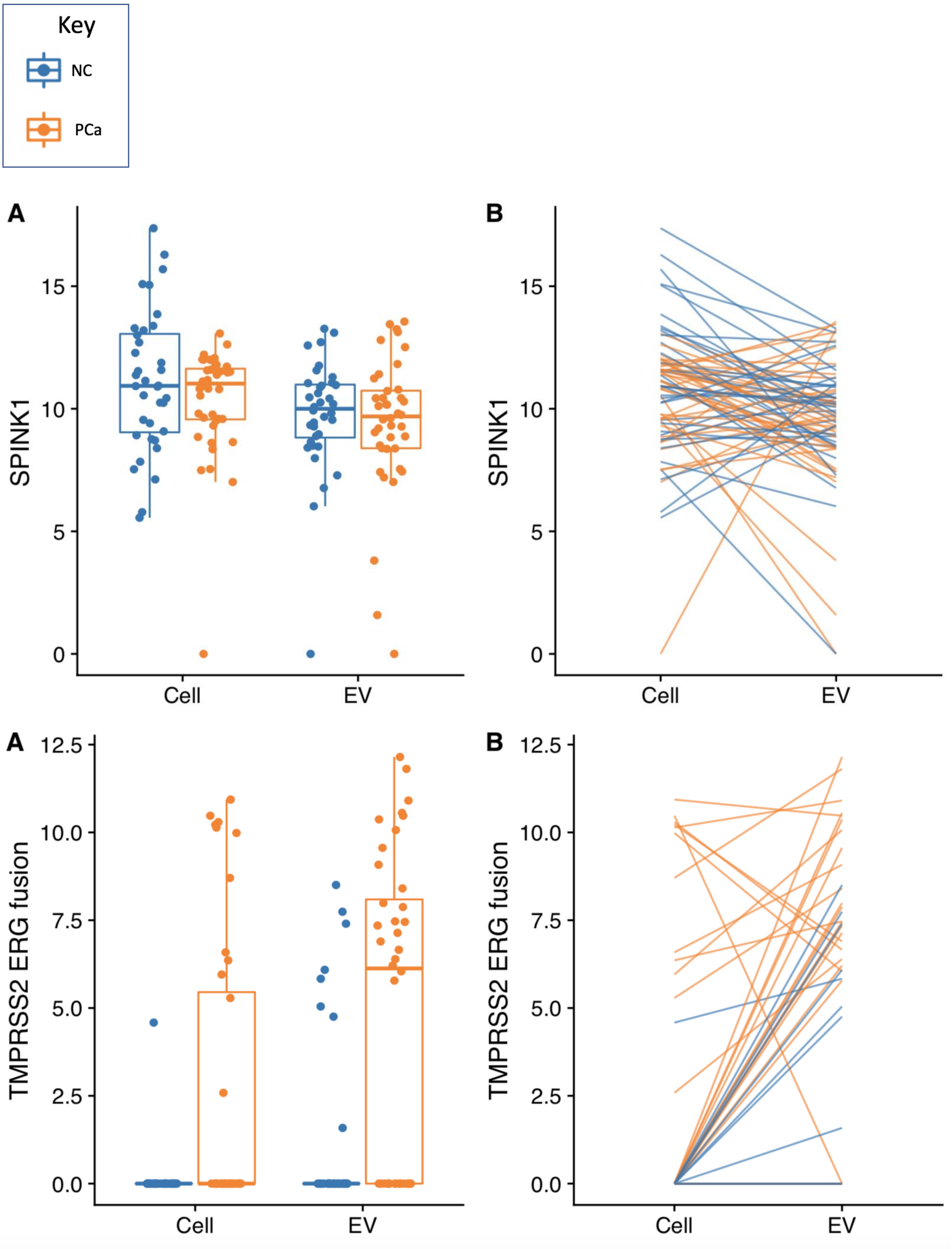
Key
NC
PCa
A
B
SPINK1
15
10
5
0
Cell
EV
TMPRSS2 ERG fusion
12.5
10.0
7.5
5.0
2.5
0.0

Figure S2: Comparison of NanoString data and qRT-PCR data for 5 gene-transcripts

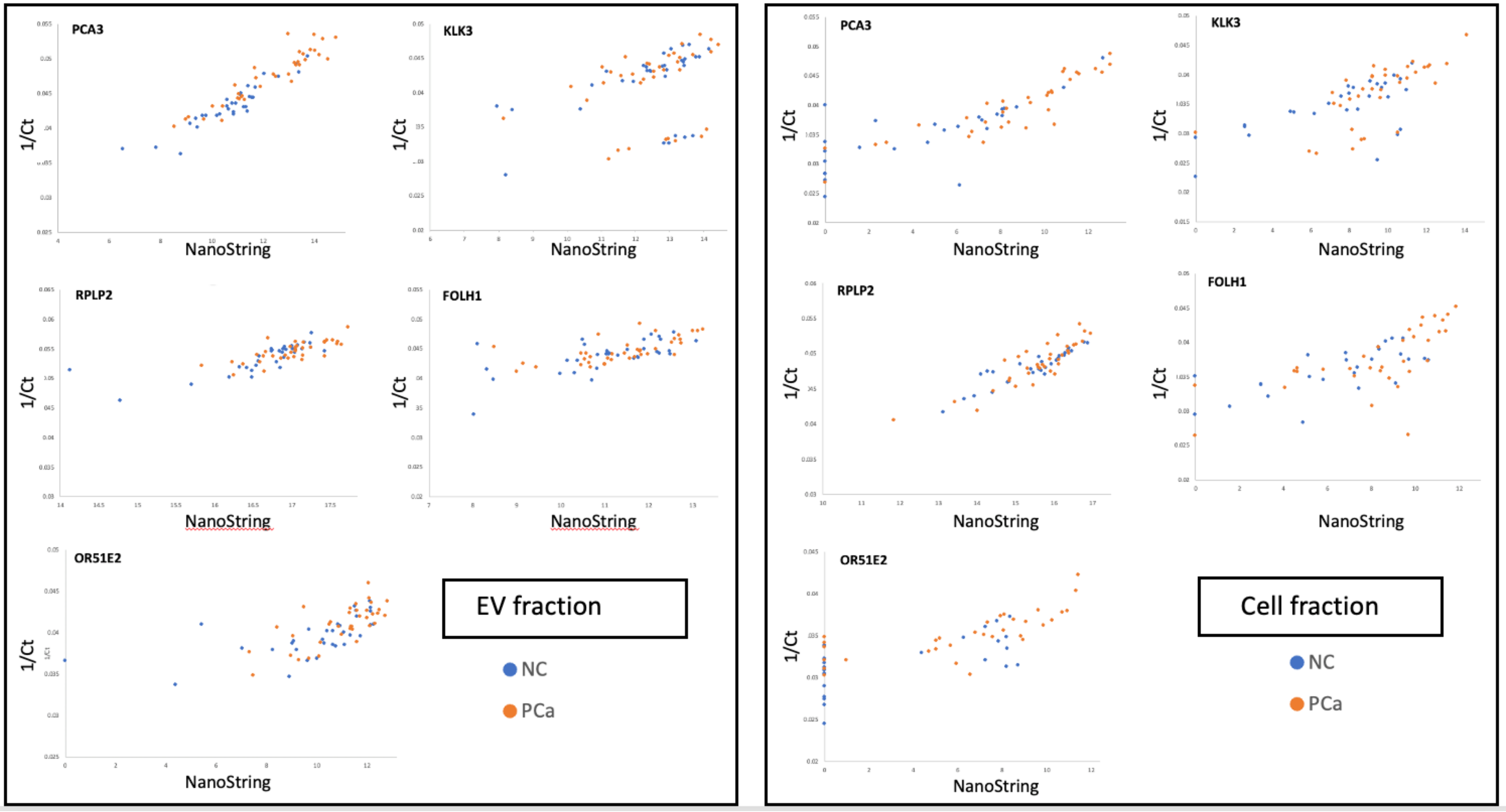

Figure S2: Comparison of NanoString data and qRT-PCR data *(EV n=71, Cell-sediment n=66)* for 5 gene-transcripts (*RPLP2*, *FOLH1*, *OR51E2, PCA3* and *KLK3)*. NanoString data is presented as log2, qRT-PCR data is presented as 1/Ct. blue dots are non-cancer (NC), orange dots are prostate cancer (PCa) samples. Data from urine EVs are in the left panel, and data from urine cell-sediment (Cell) are in the right panel. Respective Spearman correlation coefficients for *RPLP2, FOLH1, OR51E2* and *PCA3*: $r = 0.79$; $r = 0.68$; $r = 0.65$, $r = 0.94$, all $p<0.00001$) and Cell ($r = 0.86$; $r = 0.71$; $r = 0.77$; $r = 0.88$, all $p<0.00001$). For *KLK3*: when all the data was included there was a large correlation in Cell ($r = 0.70$, $p<0.00001$) but in EV samples the correlation was reduced ($r = 0.51$, $p = 0.0017$) (See Main Text and Discussion). Correlation of *KLK3* NanoString and RT-PCR data without these 13 samples provided r values of >0.85 for both fractions.
